# Investigation of Alumina-Doped *Prunus domestica* Gum Grafted Polyaniline Epoxy Resin for Corrosion Protection Coatings for Mild Steel and Stainless Steel

**DOI:** 10.3390/polym14235128

**Published:** 2022-11-25

**Authors:** Muhammad Kamran, Anwar ul Haq Ali Shah, Gul Rahman, Salma Bilal, Philipp Röse

**Affiliations:** 1 Institute of Chemical Sciences, University of Peshawar, Peshawar 25120, Khyber Pakhtunkhwa, Pakistan; 2 National Centre of Excellence in Physical Chemistry, University of Peshawar, Peshawar 25120, Khyber Pakhtunkhwa, Pakistan; 3 Karlsruhe Institute of Technology (KIT), Institute for Applied Materials-Electrochemical Technologies (IAM-ET), 76131 Karlsruhe, Germany

**Keywords:** PANI composite, corrosion protection, mild steel, Tafel analysis, epoxy resin, PDG, Al_2_O_3_, corrosion inhibition efficiency

## Abstract

Eco-friendly inhibitors have attracted considerable interest due to the increasing environmental issues caused by the extensive use of hazardous corrosion inhibitors. In this paper, environmentally friendly PDG-g-PANI/Al_2_O_3_ composites were prepared by a low-cost inverse emulsion polymerization for corrosion inhibition of mild steel (MS) and stainless steel (SS). The PDG-g-PANI/Al_2_O_3_ composites were characterized by different techniques such as X-ray diffraction (XRD), UV/Vis, and FTIR spectroscopy. XRD measurements show that the PDG-g-PANI/Al_2_O_3_ composite is mostly amorphous and scanning electron micrographs (SEM) reveal a uniform distribution of Al_2_O_3_ on the surface of the PDG-g-PANI matrix. The composite was applied as a corrosion inhibitor on mild steel (MS) and stainless steel (SS), and its efficiency was investigated by potentiodynamic polarization measurement in a 3.5% NaCl and 1 M H_2_SO_4_ solution. Corrosion kinetic parameters obtained from Tafel evaluation show that the PDG-g-PANI/Al_2_O_3_ composites protect the surface of MS and SS with inhibition efficiencies of 92.3% and 51.9% in 3.5% NaCl solution, which is notably higher than those obtained with untreated epoxy resin (89.3% and 99.5%). In particular, the mixture of epoxy/PDG-g-PANI/Al_2_O_3_ shows the best performance with an inhibition efficiency up to 99.9% on MS and SS. An equivalent good inhibition efficiency was obtained for the composite for 1M H_2_SO_4_. Analysis of activation energy, formation enthalpy, and entropy values suggest that the epoxy/PDG-g-PANI/Al_2_O_3_ coating is thermodynamically favorable for corrosion protection of MS and exhibits long-lasting stability.

## 1. Introduction

Many metals and steels are very susceptible to corrosion. As a result, huge quantities of metals are scrapped every year, causing enormous losses to the global economy. Protecting metals from corrosion is therefore essential to mitigate the limitations in industrial use. In this context, organic coatings have attracted much attention due to their excellent low-cost production, improved corrosion inhibition, excellent mechanical properties, high functionality, and environmental friendliness [1]. Among organic coatings, resins are widely used, especially water-based epoxy resins, which are applied for anti-corrosion coatings due to their excellent adhesion, corrosion-inhibiting properties, and mechanical durability [2]. Mechanical damage in organic coatings such as age-related brittleness, fractures, and external mechanical stress can compromise the integrity of the coating and reduce its long-term usability. The damage is difficult to detect and repair, and thus provides opportunities for oxygen and water to penetrate through cracks and cause corrosion [3]. The development of self-healing coatings is key to repairing damaged surfaces spontaneously and thus protecting metal surfaces [4,5,6,7,8,9,10]. Currently, two types of self-healing coatings are used: First, coatings in which self-healing is achieved by reversible formation of hydrogen bonds with other functional groups. Second, coatings in which external components, such as hollow fibers or microcapsules containing repair agents, are incorporated into the coating. Once the coating is damaged, these repair agents are released to fill and repair the cracks, initiating self-healing [11,12,13]. Tao et al. developed a very efficient coating by adding silica nanoparticles (SNPs) and microcapsules to an aqueous epoxy resin. The corrosion current density was greatly affected by an increase or decrease in microcapsules and SNPs [8]. Ma et al. reported on epoxy resin coatings containing double-walled microcapsules to achieve self-healing ability with great success [14]. However, due to its high rigidity, low shock resistance, and hygroscopic properties, the use of epoxy resin as an anticorrosion coating has become questionable. The disadvantages can be compensated by using composites of different materials [15].

Nowadays, conductive polymers have multiple applications such as in energy storage, wastewater purification [16], sensing, and adsorption [17] and corrosion protection [18]. Among them, polyaniline (PANI) is widely used as a corrosion protection material due to its excellent physicochemical properties such as easy synthesis, redox reversibility, and good environmental compatibility [18]. Therefore, the incorporation of PANI into epoxy coatings has proven to be an excellent coating material for the corrosion protection of steel parts [19]. However, the environmental benefits of PANI, such as the introduction of materials from plants with excellent insulating and mechanical properties, have been little explored. These green materials are natural gums used alone or in combination with conductive polymers and metal oxides as coating materials for the corrosion protection of metals [20]. Natural gums are usually carbohydrates with electron-rich hydroxy functional groups that assist adhesion by forming a covalent bond with metal surfaces, thus forming an insulating layer, and reducing corrosion [21,22]. On the other hand, metal/metal oxide nanoparticles are added to the epoxy coating to increase the durability in harsh corrosive environments. Various metal oxides such as Fe_2_O_3_, ZnO, TiO_2_, and ZrO_2_ are commonly used in epoxy coatings to improve their corrosion protection [23].

In this paper, we report on the synthesis of PDG-g-PANI/Al_2_O_3_ composite structures and their application in a corrosion protection system consisting of epoxy/PDG-g-PANI/Al_2_O_3_, which meets the requirements for an optimal and sustainable coating as a corrosion protection coating for stainless steel and MS in saline and acidic environment. Epoxy resin provides strong adhesion to the metal surface, high tensile strength, excellent insulation performance, and high thermal stability of the coating. PDG in the composite improved the insulating, anti-corrosive, and mechanical properties of a green approach to PANI. The introduction of Al_2_O_3_ into the polymer blend fills pores caused by the evaporation of the organic solvents during the drying process. Likewise, as a result, the composite possesses self-healing properties, which allow it to be used under highly corrosive conditions. Thus, the resulting epoxy/PDG-g-PANI/Al_2_O_3_ coating showed promising corrosion protection properties for MS and SS in acidic and saline media.

## 2. Materials and Methods

### 2.1. Materials

Aniline (C_6_H_5_NH_2_, Sigma Aldrich) was distilled two times under reduced pressure and was stored at low temperature prior to use. *Prunus domestica* gum (PDG) was collected from local gardens of district Peshawar, Pakistan, washed with distilled water, and was dried at room temperature for 14 days. The dried PDG was crushed and grinded to powder form. Diesel was purchased from Pakistan state oil (PSO, Karachi, Pakistan), with a boiling range from 160 to 366 °C, specific gravity of 0.828, and viscosity and 3.11 cst at 20 °C, respectively, and was used as received. Aluminum oxide (Al_2_O_3_, Merck), benzoyl peroxide (C_14_H_10_O_4_, Merck), dodecylbenzenesulfonic acid (C_12_H_25_C_6_H_4_SO_3_H, DBSA, Acros organic), chloroform, *n*-hexane, methanol, ethanol, propanol, 2-butanol, acetone, and *N*-methyl-2-pyrrolidone (NMP) were purchased from (Sigma Aldrich, St. Louis, MI, USA). Magic^®^ epoxy glue (resin 100 g, hardener 80 g, 7 Mart Ltd., Punjab, Pakistan) with a mean viscosity of 16.500 cps and mean specific gravity of 1.10 was used without further treatment. Doubled distilled water was used for all the experiments.

### 2.2. Synthesis of PDG Grafted Polyaniline/Aluminium Oxide (PDG-g-PANI)/Al_2_O_3_ Composites

For the synthesis of the (PDG-g-PANI)/Al_2_O_3_ composite, 30 mL of distilled water was placed in a flask and then 0.1 g of Al_2_O_3_ powder was added and dispersed by ultrasonication for 30 min. Then, 0.1 g PDG powder was added under constant stirring. After 30 min, 1 mL of DBSA, 30 mL diesel and 0.4 mL of aniline were added to the suspension. For initializing polymerization, 1 g of BPO oxidant was added and the whole mixture was stirred at room temperature for 24 h. Over this time, a dark green product was precipitated. The product was washed with distilled water several times to remove water soluble impurities; a proper amount of acetone was added to disperse the product. The dispersion was poured into a ceramic tray and dried at room temperature. After drying, the product was washed with *n*-hexane for the removal of non-polar impurities. The obtained product was dried at 40 °C for 2 h.

### 2.3. Composite Characterisation Methods

UV/Vis spectra of PGD-g-PANI/Al_2_O_3_ were collected in chloroform in a spectral range from 200 to 1100 nm with a Lamda 1050 spectrometer (Waltham, MA, USA). Fourier transformed infrared spectroscopy (FTIR) was performed on an Affinity-1S FT-IR spectrometer from Shimadzu (Kyoto, Japan), scanning over an effective range of 400 to 4000 cm^−1^, with a 2 cm^−1^ resolution. X-ray diffraction (XRD) analysis was carried out using an X-ray diffractometer from Rigaku (Tokyo, Japan) with Cu Kα radiation (λ = 1.54 A) at 40 kV and 35 mA current with 2θ ranging from 10° to 80°, step width of 0.0164°, and a step rate of 1 s^−1^. Imaging and structural analysis of the composites were performed using a scanning electron microscope (JSM-5910, JEOL, Tokyo, Japan).

### 2.4. Preparation of the Anti-Corrosion Coatings

Epoxy resin and hardener (1:1 *v*/*v*, total 0.5 g) were thoroughly mixed and applied together. Then PDG-g-PANI/Al_2_O_3_ (4.5 wt%) was suspended in 5 mL chloroform and mixed with the epoxy adhesive. The mixture was kept at room temperature for 30 min. Steel electrodes were polished using different sized sandpapers with 120 to 800 grit sizes and smoothed with a polishing pad containing alumina oxide suspension (0.05 μm), followed by washing with acetone, and double distilled water. Then the mixture 1 mL of the solution was drop casted on the MS or SS surface and dried at room temperature in an open atmosphere.

### 2.5. Electrochemical Characterization

Electrochemical characterizations of PDG-g-PANI/Al_2_O_3_ samples were carried out by using Reference 600 ZRA potentiostat/galvanostat (Gamry, Warminster, PA, USA), in three electrode assembly and in 1 M H_2_SO_4_ solution using gold sheet as the working electrode, gold wire as the counter, and saturated calomel electrode as the reference electrode. Cyclic voltammetry measurements were performed in the potential range −0.2 to 0.9 V at scan rates ranging from 10 mV s^−1^ to 500 mV s^−1^.

Anti-corrosion analysis of the composite was performed in a corrosive solution of 3.5% NaCl and 1M H_2_SO_4_. Corrosion experiments for blank and coated MS and SS disks were performed in a customized cell with three electrodes using the Reference 600 ZRA potentiostat/galvanostat (Gamry, Warminster, PA, USA). MS and SS disks were used as the working electrodes, saturated calomel electrodes (SCE) as the reference electrodes, and stainless-steel plates as the counter electrodes. Cathodic/anodic potentiodynamic polarization measurements were performed and corrosion kinetic parameters such as *i_corr_* (corrosion current density), *E_corr_* (corrosion potential) and the corrosion rate C_R_ (mm/year) for MS and SS were calculated by extrapolating Tafel plots using Gamry Echem Analyst software. The inhibition efficiency was determined by using Equation (1).

(1) $$Inhibition\;efficiency\;\left(I.E.\right)\%=1-\frac{i_{corr}}{i_{corr}^{0}}\times 100$$

where $i_{corr}^{0}$ is the corrosion current density of blank steel electrodes, while $i_{corr}$ is the corrosion current density of coated steel electrodes.

The corrosion rate was calculated using the weight loss per time. To ensure an accurate measurement, two comparable electrodes were polished, then thoroughly cleaned with ethanol, acetone, and distilled water, dried in an oven to constant weight and weighed. Then, one of the electrodes was coated with the composite and tested for corrosion resistance under saline and acidic conditions. An uncoated electrode was used as a reference sample. The corrosion rate was then calculated according to Equation (2):

(2) $$Corrosion\;rate=\frac{W}{D\cdot A\cdot t}$$

where *W* is weight loss in g, *D* is density in g cm^−3^, *A* is area in cm^2^, and *t* is time of exposure in seconds (s).

## 3. Results and Discussion

### 3.1. Solubility Study

**Figure 1:**
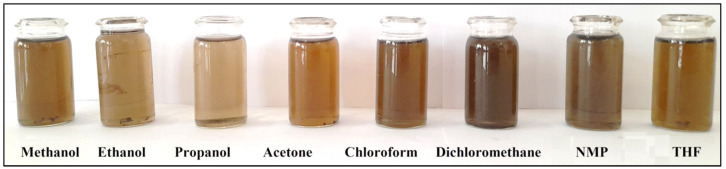
Solutions of PDG-g-PANI/Al_2_O_3_ in various common organic solvents.

The composite solubility is an important property from an application point of view since both the processability and the resistance in different areas of the application are key criteria. To investigate the solubility of the PDG-g-PANI/Al_2_O_3_ composite, various organic solvents were explored (Figure 1). It was found that the composite is soluble in alcohols, especially methanol and ethanol/propanol, which is due to the presence of polar components such as –NH and –OH, which are responsible for the solubility in polar solvents. Such solubility can be attributed to the formation of hydrogen bonds between composites and alcohols [20]. The same phenomenon occurred when the composite approached NMP and THF, albeit these solvents differ significantly from alcohols. The presence of the amide and ether functional groups in NMP as well THF may form hydrogen bonds with −OH of PDG and −NH of PANI in the composite particles.

The solubility of the composite in dichloromethane can be attributed to a dipole-dipole interaction, since the composite is doped with DBSA, which contains both a nonpolar alkyl chain and a polar sulfonate group. The solubility of the composite in nonpolar solvents such as acetone and chloroform are due to the nonpolar interaction between the solvent and the nonpolar components present in the composite [24,25].

### 3.2. UV/Visible Spectroscopy

**Figure 2:**
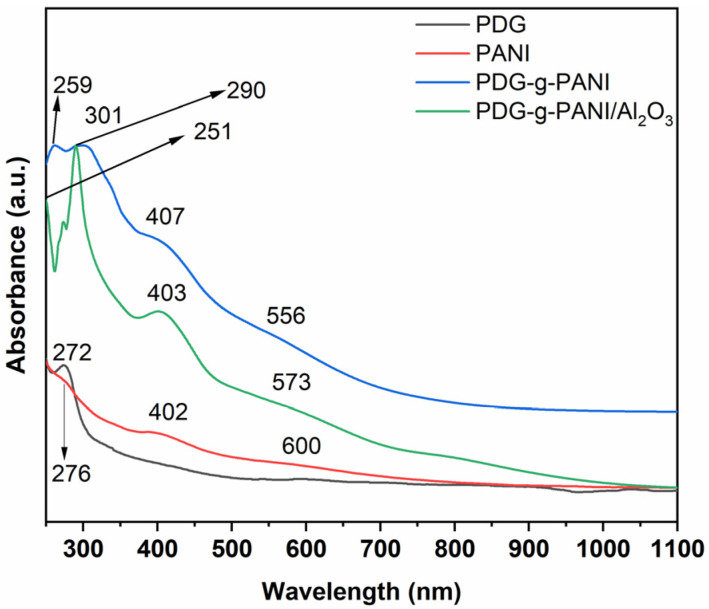
UV/Vis spectra of PDG, PANI, PDG-g-PANI, and PDG-g-PANI/Al_2_O_3_.

The absorption spectra of neat PDG and PANI, PDG-g-PANI, and PDG-g-PANI/Al_2_O_3_ in chloroform were recorded by UV/Vis (200–1100 nm, see Figure 2). The arbinogalactan components of PDG showed absorption at 272 nm. The spectrum of PANI revealed peaks at 276 nm, 402 nm, and a broadened characteristic at 600 nm. These peaks were due to the π-π*-transition of the benzoid ring, the polaron π*-transition of the quinoid ring, and the π-polaron transition [26]. The PDG-g PANI spectrum exhibited peaks at 259 nm, 301 nm, 407 nm, and a broadened feature at 556 nm. The region at 556 nm is attributed to the π-polaron transition, while the peak at 407 nm is attributed to the polaron π*-transition of the quinoid ring. Peaks at 301 nm and 261 nm are attributed to the π-π*-transition of the benzoid ring and the arabinogalactose component of PDG, respectively [20]. The UV/Vis spectra of PDG-g-PANI/Al_2_O_3_ gave three sharp peaks at 251 nm, 290 nm, and 403 nm, while there was one broad peak at 573 nm. The occurrence of sharp peaks is due to the incorporation of Al_2_O_3_ into the PDG-g-PANI backbone. The peak at about 251 nm is attributed to the arbinogalactan components of PDG, while the sharp peak at 290 nm originates from the benzoid ring of PANI. A clear blue shift is observed in both peaks, which can be attributed to the incorporation of Al_2_O_3_ into the polymer chain. The peak at about 407 nm is attributed to the polaron-π* transition of the quinoid ring, while a broad absorption peak at 573 nm is attributed to the exiton transition in the composite. All these peaks indicate the formation of PDG-g-PANI/Al_2_O_3_.

### 3.3. FTIR Analysis

**Figure 3:**
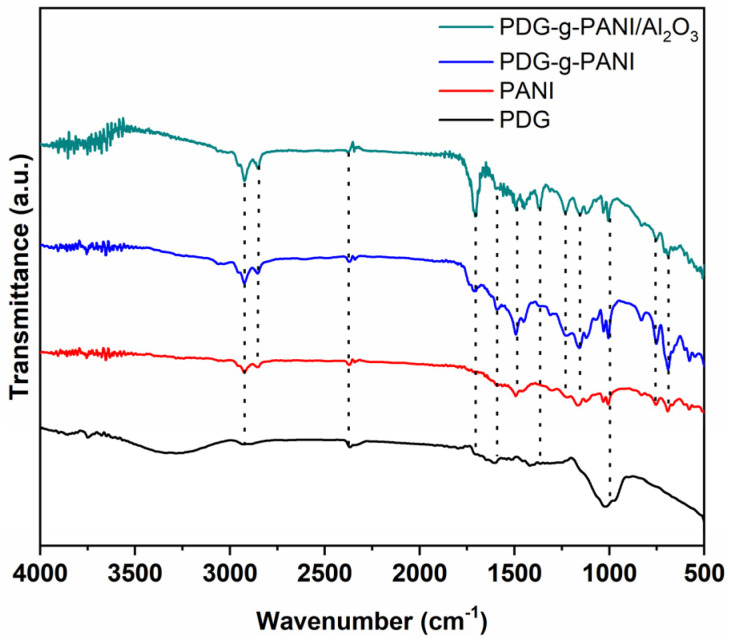
FTIR spectra of the PDG, PANI, PDG-g-PANI, and PDG-g-PANI/Al_2_O_3_.

Figure 3 shows the FTIR spectra of PDG, PANI, PDG-g-PANI, and PDG-g-PANI/Al_2_O_3_, and the peak assignments concerned are given in Table 1 [20]. In the spectrum of PDG-g-PANI/Al_2_O_3_, the bands at 2917 cm^−1^ and 2847 cm^−1^ are due to coupled stretching vibrations of the aliphatic C-H of PDG and C-H of aniline in the polymer chain. The typical peak of -COOH occurring at 1716 cm^−1^ due to the stretching vibration of -C=O confirms the grafting of PDG into the polymer chain. The peak at 1490 cm^−1^ and 1451 cm^−1^ is assigned to the C=C stretching of quinolines and benzene rings, respectively [27]. The shift in quinoid stretching indicates the doping of the polymer chain with metal oxide. The peak at 1372 cm^−1^ is due to C-N stretching, while the peak at about 1003 cm^−1^ is assigned to C-H stretching vibration. The peak at 826 cm^−1^ was due to out-of-plane C-H deformation. The band at 753 cm^−1^ is assigned to the C-H bending vibration, while the occurrence of the peak at 1003 cm^−1^ is due to -SO_3_H of DBSA, confirming the doping of DBSA into the polymer chain [26]. The peak at 689 and 580 cm^−1^ is attributed to Al_2_O_3_, confirming the incorporation of Al_2_O_3_ into the PDG-g-PANI chain [28].

### 3.4. X-ray Diffraction

**Figure 4:**
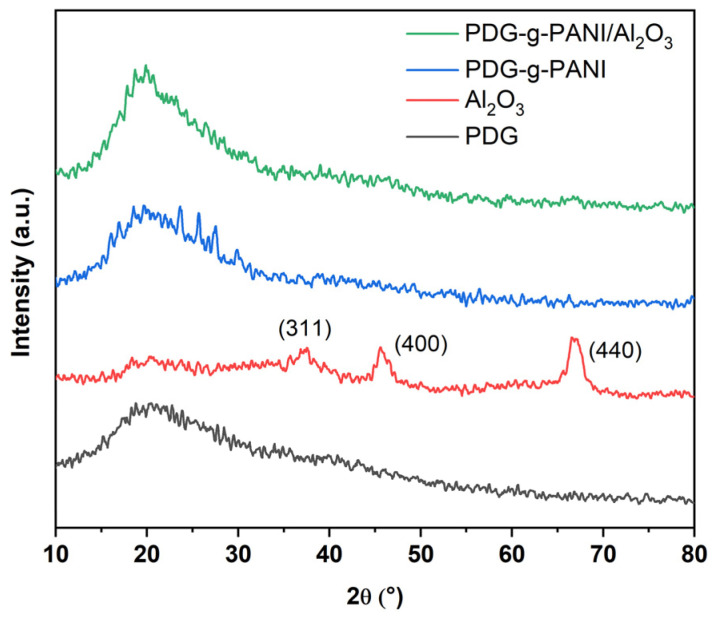
XRD pattern of pristine Al_2_O_3_, PDG, PDG-g-PANI, and PDG-g-PANI/Al_2_O_3_.

The XRD pattern of pristine Al_2_O_3_, PDG, PDG-g-PANI, and PDG-g-PANI/Al_2_O_3_ is shown in Figure 4. The XRD pattern of Al_2_O_3_ exhibits characteristic diffraction peaks at 37.5°, 45.7°, 66.6° corresponding to (311), (400), and (440) lattice planes of γ-Al_2_O_3_ [29]. Both the pristine PDG and the PDG-g-PANI show broad peaks in the range of 17° and 21°, indicating the amorphous nature of the natural rubber and the polymer. The incorporation of Al_2_O_3_ in the PDG-g-PANI matrix as a nanofiller did not change the mainly amorphous structure [30].

### 3.5. Energy Dispersive X-ray *Analysis*

**Figure 5:**
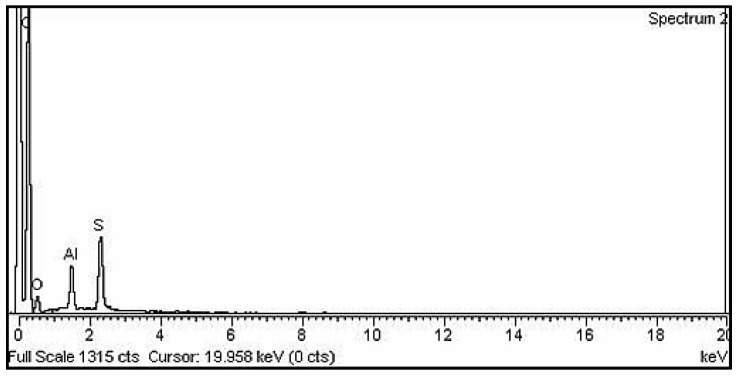
EDX spectrum of PDG-g-PANI/Al_2_O_3_.

Energy dispersive X-ray (EDX) analysis was performed to verify the incorporation of Al_2_O_3_ into the PDG-g-PANI matrix. The result is shown in Figure 5, and the corresponding elemental composition is listed in Table 2. The high carbon content originates from aniline and PDG in the composite, since it is the main content in both compounds. The sulfur content of 4.65 wt% indicates an efficient doping of the PANI backbone by DBSA, while the presence of alumina confirms the incorporation of Al_2_O_3_ into the polymer matrix.

### 3.6. Scanning Electron Microscopy Analysis

**Figure 6:**
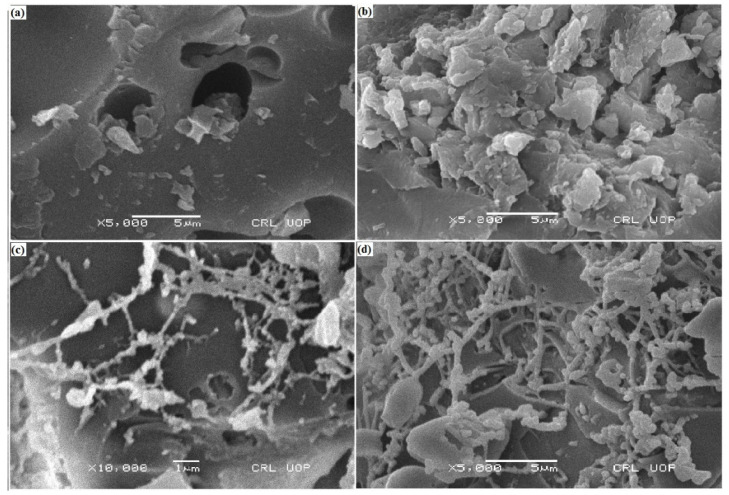
SEM micrographs of (**a**) PANI (**b**) PDG (**c**) PDG-g-PANI, and (**d**) PDG-g-PANI/Al_2_O_3_.

SEM images of PDG, PANI, PDG-g-PANI, and PDG-g-PANI/Al_2_O_3_ are shown in the Figure 6. PANI exhibits a porous morphology while the PDG shows irregular surface characteristics. The image of PDG-g-PANI displays a porous surface of PANI having a PDG network surrounding the porous network of PANI [20]. For PDG-g-PANI/Al_2_O_3_ it can be seen that all three components including PANI, PDG, and Al_2_O_3_ particles are present in the composite. PANI forms the principal matrix surrounded by the fibrous network of PDG. It is noticed that Al_2_O_3_ particles are distributed along the whole network of the composite. The images show that PDG and alumina particles block the pores in the matrix of the polymer and thus restrict the movement of corrosive ions to reach the metal surface, protecting it from corrosion. This results in an increase in corrosion protection capability. 

### 3.7. Cyclic Voltammetry

**Figure 7:**
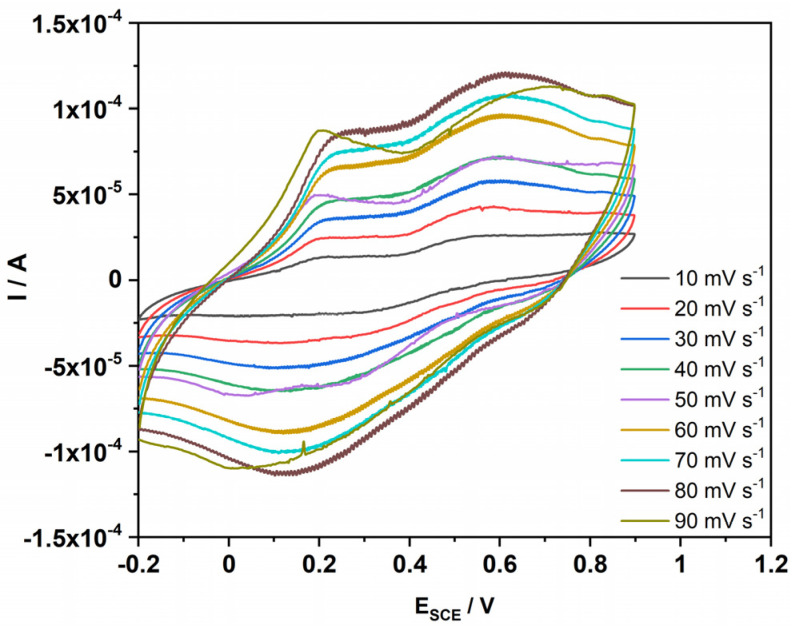
Cyclic voltammogram of PDG-g-PANI/Al_2_O_3_ at different scan rates obtained in 1 M aqueous H_2_SO_4_ solution.

Figure 7 displays the cyclic voltammogram of PDG-g-PANI/Al_2_O_3_ at different scan rates from 10 to 90 mV s^−1^ obtained in 1 M aqueous H_2_SO_4_ solution in the potential range of −0.2 to 0.9 V. The CV exhibits the two characteristic oxidation and reduction peaks of PANI. The first peak at E_SCE_ = 0.19 V is assigned to the conversion of neutral leucoemeraldine to the partially oxidized emeraldine form of PANI, while the second oxidation peak at 0.60 V is attributed to the redox transition of the emeraldine to pernigraniline. In the reverse scan, the conversion of pernigraniline to emeraldine is at 0.68 V, while the peak at −5 mV shows the conversion of the emeraldine form of PANI back to the fully reduced leucoemeraldine [31]. The observed redox peaks are particularly associated with the PANI composite, indicating that the polymerization of aniline in the presence of PDG and Al_2_O_3_ nanoparticles generates an electroactive reversible composite.

### 3.8. Investigation of the Corrosion Behavior

#### 3.8.1. Potentiodynamic Polarization Study of Mild Steel in NaCl Solution

**Figure 8:**
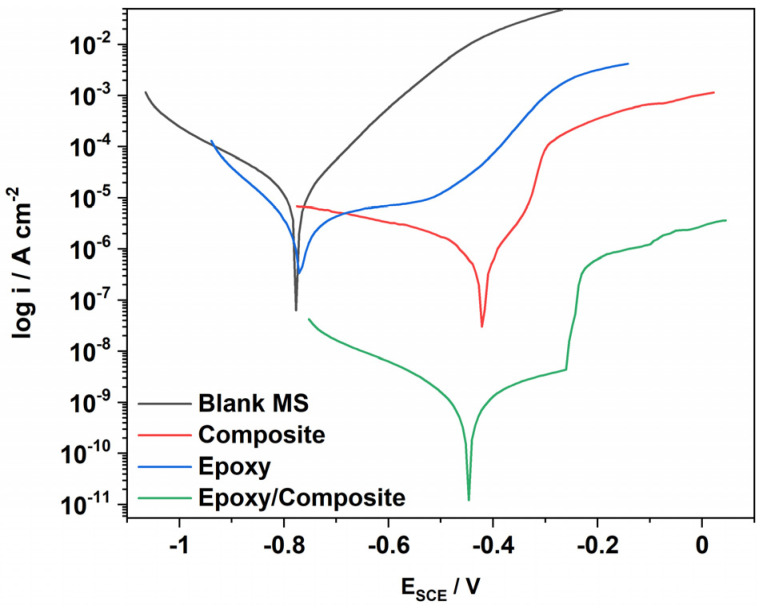
Tafel plot of uncoated, PDG-g-PANI/Al_2_O_3_, Epoxy, and Epoxy/PDG-g-PANI/Al_2_O_3_ coated MS in 3.5% NaCl.

Potentiodynamic polarization measurement is a suitable analysis technique for corrosion protection coating systems. Tafel curves of the uncoated, bare composite, bare epoxy, and epoxy/composite coated mild steel (MS) immersed in 3.5 wt% NaCl solution are shown in Figure 8. The corrosion kinetic parameters calculated from polarization curves by Tafel extrapolation are presented in Table 3. The corrosion current density and corrosion potential of uncoated MS are 20.2 μA and −777 mV, with a corrosion rate of 9.224 m/year. The PDG-g-PANI/Al_2_O_3_ coating on MS reduces the current density to 1.56 μA, thus shifting the corrosion potential to −420 mV and reducing the corrosion rate to 0.711 m/year with an inhibition efficiency of 92.3%. The pristine epoxy coating on MS also shows an inhibition efficiency of 89.3%. Incorporation of the PDG-g-PANI/Al_2_O_3_ composite into the epoxy coating inhibits the surface of MS by two simultaneous effects: (1) the inorganic Al_2_O_3_ particles in polymer coating provides a physical barrier for the corrosive environment from reaching the metal surface by filling the nanopores and micropores in the coating [32,33]; (2) the PDG-g-PANI matrix enhances the corrosion protection of epoxy coating due to its electrical conductivity and the formation of a strong protective oxide layer on the MS surface [34]. The lowest current density and inhibition efficiency of 99.9% indicates that the presence of Al_2_O_3_ has a great impact on epoxy/composite coating.

#### 3.8.2. Corrosion Study of Stainless Steel in NaCl Solution

**Figure 9:**
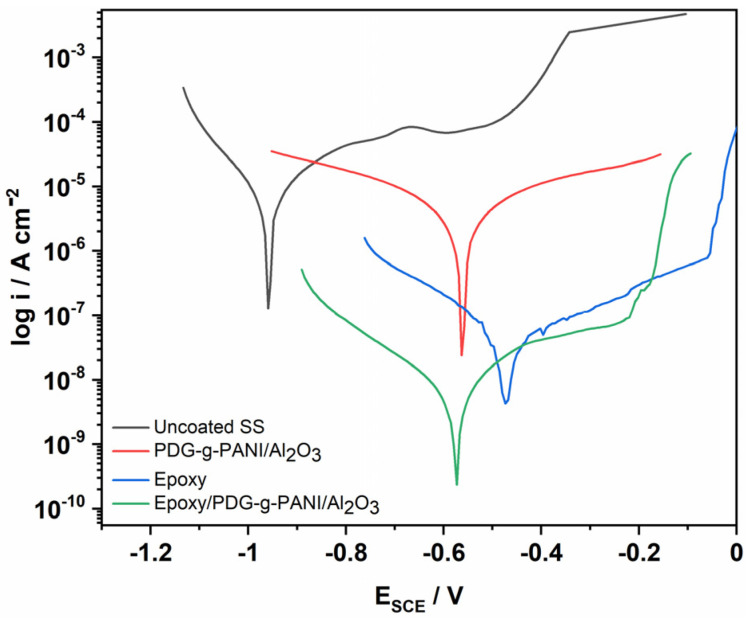
Tafel plot of uncoated, PDG-g-PANI/Al_2_O_3_, Epoxy, and Epoxy/PDG-g-PANI/Al_2_O_3_ coated SS in 3.5% NaCl.

The behavior of uncoated and composite, epoxy and epoxy/composite coated SS were also tested in 3.5% NaCl solution and the results are shown in Figure 9 and Table 4. Uncoated SS shows a corrosion current density of 11.9 μA and −960 mV for the corrosion overpotential, with a corrosion rate of 5.42 m/year. With coating, the corrosion rate decreases to 2.616 m/year with a lowered corrosion current density of 5.72 μA, a positive shift of corrosion potential value of −563 mV, and an inhibition efficiency of 52%. However, the PDG-g-PANI anti-corrosion behavior is still moderate, which can be attributed to the low adhesion on SS, which allows the salt solution to penetrate the surface of the steal and cause corrosion. For improvement, the composite was integrated into an epoxy matrix with strong adhesion and mechanical durability. The epoxy/composite coating on SS shows an excellent anti-corrosion behavior with a corrosion rate of 0.0051 m/year and efficiency of 99.9%.

#### 3.8.3. Potentiodynamic Polarization Study of Mild Steel in H_2_SO_4_ Solution

**Figure 10:**
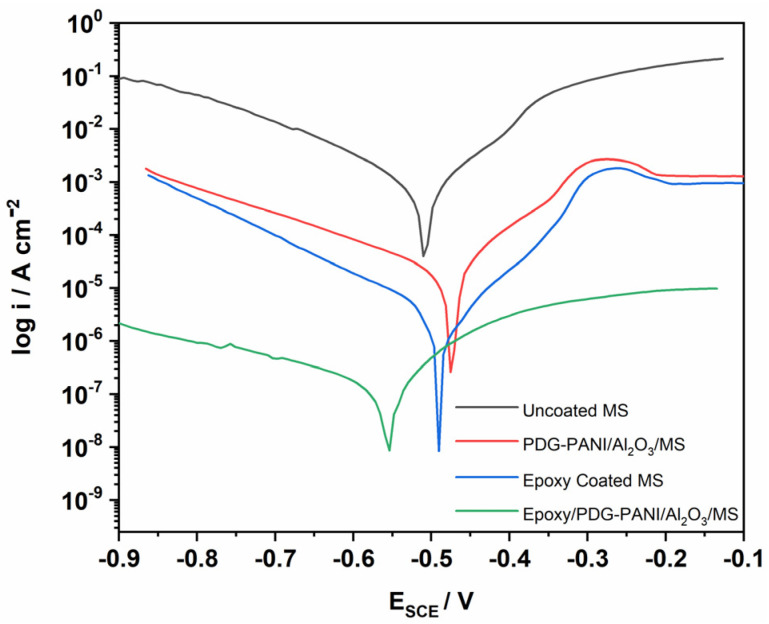
Tafel plot of uncoated, PDG-g-PANI/Al_2_O_3_, Epoxy, and Epoxy/PDG-g-PANI/Al_2_O_3_ coated MS in 1 M H_2_SO_4_.

The corrosion protection ability of PDG-g-PANI/Al_2_O_3_, pristine epoxy, and the blend of epoxy with PDG-g-PANI/Al_2_O_3_ on MS were investigated in 1 M H_2_SO_4_ and were compared with uncoated MS (Figure 10 and Table 5). The results of the corrosion current and corrosion potential for uncoated MS are 1480 μA and −508 mV. By coating PDG-g-PANI/Al_2_O_3_ on MS, the corrosion current density decreases to 11.80 μA and E_corr_ is shifted to −475 mV, showing excellent corrosion protection with an inhibition efficiency of 99.2%. On the other hand, the coating of pristine epoxy on MS decreases the corrosion current to 2.05 μA, displaying an inhibition efficiency of 99.86%. However, the coating of epoxy with composite coating inhibits the surface of steel tremendously from corrosion through the synergistic effect of all components present in a single coating. The coating reduces the corrosion current density to 0.473 μA, which is almost 1.5 times lower than for the epoxy resin, revealing an enhanced corrosion protection with perfect inhibition efficiency of 99.96% and corrosion rate of 0.216 m/year. The results conclude that PDG-g-PANI/Al_2_O_3_ blended with epoxy possesses excellent behavior as a corrosion inhibition coating in strongly acidic medium.

#### 3.8.4. Potentiodynamic Polarization Study of Stainless Steel in H_2_SO_4_ Solution

**Figure 11:**
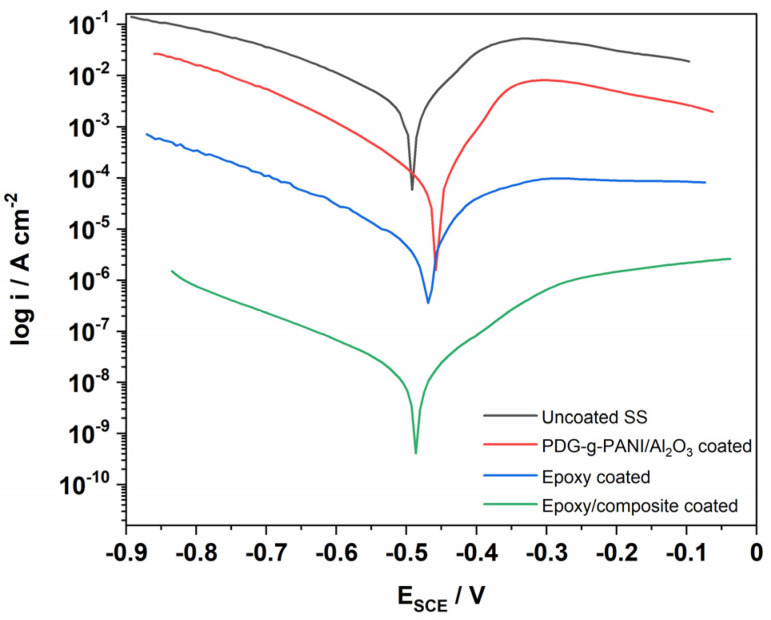
Tafel plot of uncoated, PDG-g-PANI/Al_2_O_3_, Epoxy, and Epoxy/PDG-g-PANI/Al_2_O_3_ coated SS in 1 M H_2_SO_4_.

The corrosion behavior of stainless steel in H_2_SO_4_ solution is shown in the following (Figure 11 and Table 6). Firstly, blank SS was tested towards its corrosion properties. The corrosion rate is 2157 m/year with 4.72 mA corrosion current density and −491 mV corrosion potential. When coated with PDG-g-PANI/Al_2_O_3_ the corrosion current density decreases to 96.8 μA and shifts to a corrosion potential positively with −458 mV and a decrease in corrosion rate to 44.2 m/year (97.9% inhibition efficiency). By coating pristine epoxy resin the corrosion current density and voltage drop to 6.65 μA and −470 mV with 3.03 m/year corrosion rate and 99.85% inhibition efficiency. The combination of epoxy resin and composite result in in an excellent corrosion rate of 0.014 m/year and 99.99% inhibition. The behavior of epoxy/composite coating on SS is attributed to the fact the combined epoxy/composite coating contains an optimal sticky epoxy resin with good mechanical strength, PDG with sticky behavior due to presence of electron rich functional groups and good mechanical properties, PANI with its redox reversible nature and Al_2_O_3_ with its mechanical barrier property, self-healing ability, and its resistivity [18].

#### 3.8.5. Kinetic Study in Real Outdoor Environment

**Figure 12:**
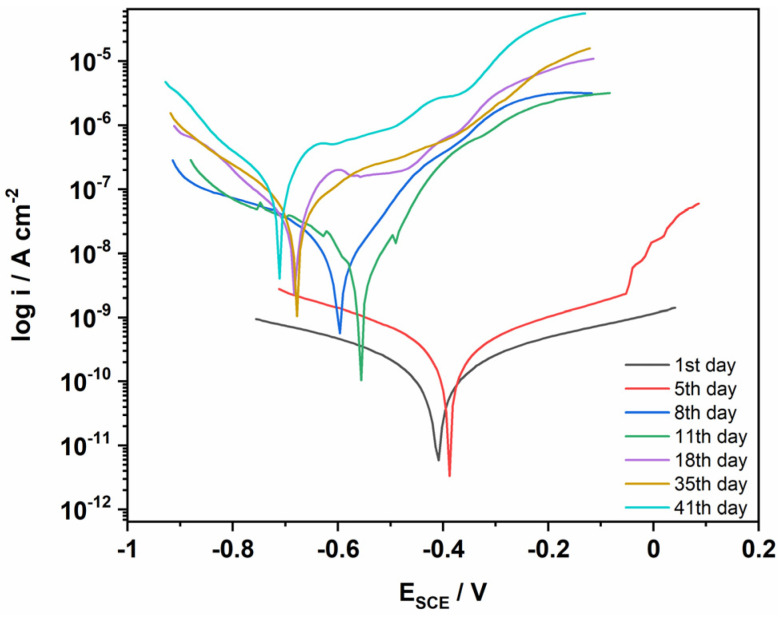
Tafel plots of Epoxy/PDG-g-PANI/Al_2_O_3_ coated MS under outdoor conditions.

The kinetic study of epoxy/composite coated MS was studied outdoors with constant treatment by salt spray after 24 h for 41 days at room temperature. The material was applied to the polished surface of the MS and dried at room temperature. After drying, the anti-corrosion behavior of the coating was tested using the potentiodynamic polarization technique in 3.5% NaCl solution. After that, the samples were stored outdoors and subjected to constant salt spray treatment every 24 h, and the corrosion ability of the coating was checked at constant time intervals. The corrosion kinetic parameters obtained from the Tafel extrapolation are shown in Table 7 and the Tafel plots are shown in Figure 12. The corrosion rate of the epoxy/composite coating decreased to a large extent after the first day of coating compared to uncoated MS. The behavior of the coating was observed at different time intervals, and it was found that the coating exhibited excellent anti-corrosion protection behavior to the surface of the MS for 41 days. After 41 days, the calculated inhibition efficiency was 99.3% with a corrosion rate of 0.0626 m/year, showing sufficient inhibition over a long period of time.

#### 3.8.6. Weight Losses during Corrosion

Quantification of the weight loss during the corrosion experiments were performed by immersing the uncoated and the epoxy/(PDG-g-PANI/Al_2_O_3_) coated MSs in a 3.5% NaCl solution for 25 days at room temperature. The corrosion rate was calculated for both the uncoated and coated electrodes based on the weight loss. The corresponding inhibition efficiencies were calculated from the corrosion rates for uncoated and coated MS; the results are shown in Table 8. The weight loss for uncoated MS was 129 mg after 25 days of immersion in 3.5% NaCl solution, whereas the weight loss of PDG-g-PANI-coated MS was 5 mg. According to the calculation, the corrosion rate of the uncoated MS was 2.41 m/year and that of the coated MS was 0.093 m/year with an inhibition efficiency of 96.1%. The results were in close agreement with the electrochemical data.

#### 3.8.7. Analysis of the Temperature Dependency on the Corrosion Rate

**Figure 13:**
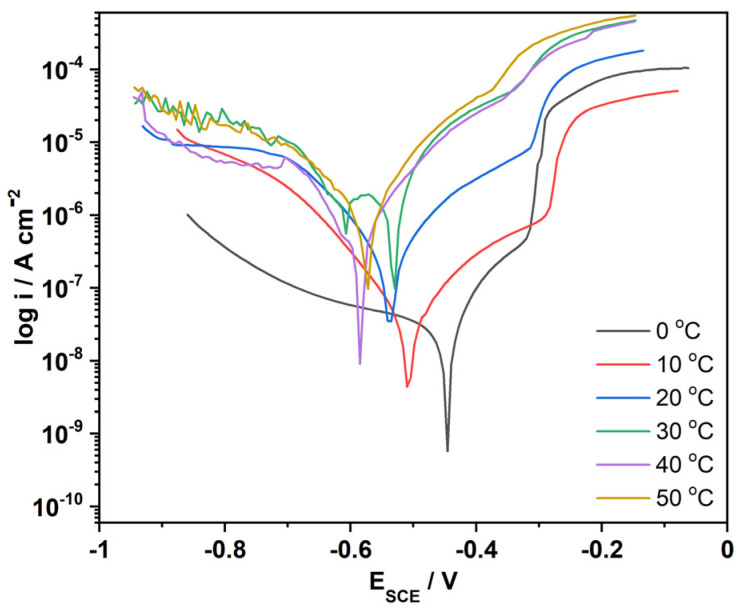
Tafel plot of Epoxy/composite coated MS at different temperatures.

For an in-depth explanation of the corrosion protection properties of the coating, the thermodynamic parameters such as activation energy, change in formation enthalpy and entropy were determined for uncoated and epoxy/PDG-g-PANI/Al_2_O_3_ coated MS. The effect of temperature on the anticorrosion reaction of the coating is complex and leads to changes in the metal surface state by desorption of the inhibitor, rapid etching, and decomposition or restructuring of the coating material. Based on the potentiodynamic polarization curve, it was observed that the corrosion rates for both coated and uncoated MS in 3.5% NaCl solutions increase with the increasing temperature (Figure 13).

**Figure 14:**
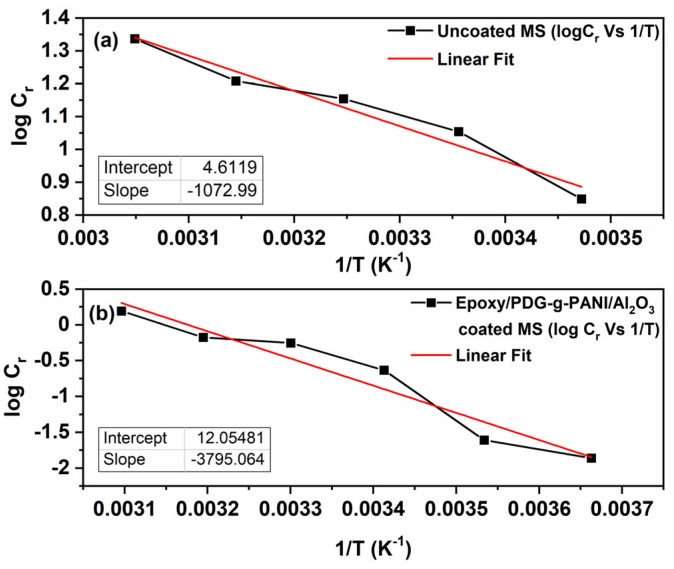
Arrhenius plots of log C_r_ versus 1/T for (**a**) uncoated MS and (**b**) Epoxy/composite coated MS.

It is well understood that the activation energy depends on temperature, which can be calculated from Arrhenius Equation (3). The logarithm of the corrosion rate *C_r_* is plotted against 1/T, as given below,

(3) $$\log C_{r}=-\frac{E_{a}}{RT}+\ln A$$

where *E_a_* is the activation energy, *R* is the universal gas constant, and *T* is the Kelvin temperature (Figure 14a,b). The *E_a_* values are calculated from the slope of the graph and are given in Table 9. The activation energy for coated MS is higher than for uncoated MS in a 3.5% NaCl solution, indicating a strong inhibition behavior through the coating by reducing the reaction rate for the corrosion process. It confirms once more the strong adsorption of the epoxy/composite coating towards a mild steel surface, and thus a superficial anti-corrosion behavior [35].

**Figure 15:**
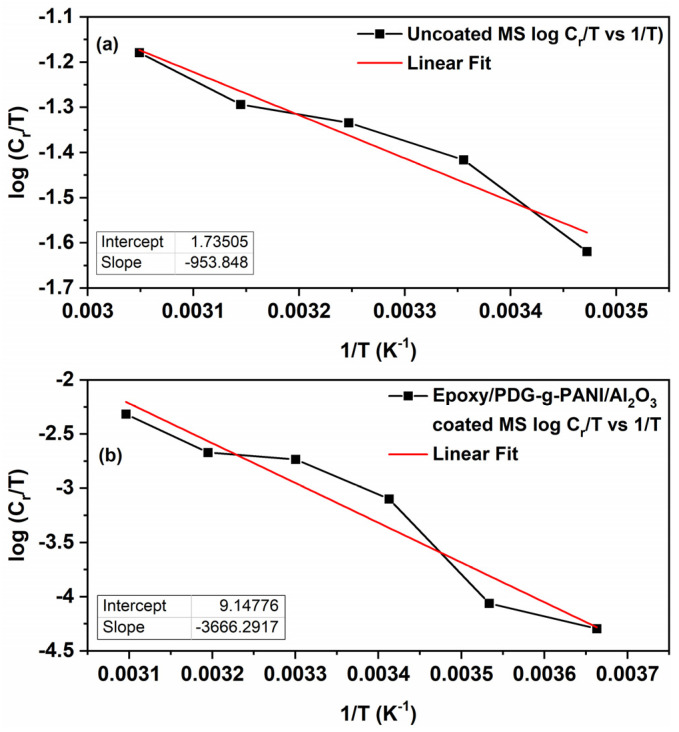
Extended Arrhenius plots of log C_r_/T versus 1/T for (**a**) uncoated MS and (**b**) Epoxy/composite coated MS.

For a closer understanding of the thermodynamics behind the anti-corrosion coating, the change of formation enthalpy ($\Delta H_{a}^{o}$) and entropy ($\Delta S_{a}^{o}$) of the activation complex between metal surface and coating material in the transition state is given by the transition-state Equation (4) [36],

(4) $$log\frac{C_{r}}{T}=\log\left(\frac{R}{Nh}\right)+\frac{\Delta S_{a}^{o}}{2.303R}-\frac{\Delta H_{a}^{o}}{2.303RT}$$

where *N* is Avogadro’s number and *h* is Planck’s constant. A plot of $log\frac{C_{r}}{T}$ over 1/T, gives a straight line with the slope = $\frac{\Delta H_{a}^{o}}{2.303R}$ and intercept $\log\left(\frac{R}{Nh}\right)+\frac{\Delta S_{a}^{o}}{2.303R}$ from which the values of enthalpy and entropy were calculated (Figure 15a,b). The values of enthalpy and entropy are given in Table 9. The higher and positive values of enthalpy for coated MS compared to uncoated MS suggested that the corrosion/dissolution process is more difficult and endothermic in coated MS. Additionally, entropy of activation $\Delta S_{a}^{o}$ for coated MS is less negative, showing an ordered arrangement of particles present in the coating on the surface of MS. This confirms the activated complex as an association step rather than a dissociation step in the rate determining step [37]. From this study it is also concluded that the epoxy/composite coating thermodynamically inhibits the surface of steel from the corrosion process as well.

## 4. Conclusions

In this work, we successfully synthesized a green composite based on PDG-g-PANI/Al_2_O_3_ by a low-cost inverse emulsion polymerization. Material characterization showed that all three components were incorporated into a stable matrix, while cyclic voltammetry proved that the redox activity of PANI was preserved. The composite was used as an anti-corrosion coating for mild and stainless steel in salty and acidic media. The corrosion kinetic parameters obtained from the Tafel analysis showed that the PDG-g-PANI/Al_2_O_3_ composite protected the surface with a high inhibition efficiency of 92.3% and 51.9% for mild and stainless steel, respectively, in a 3.5% NaCl solution, which was significantly higher than the value without coating. When additionally dispersed in epoxy resin, the combination of epoxy and PDG-g-PANI/Al_2_O_3_ showed the best performance, with an inhibition efficiency of 99.9% for both steels in saline as well as acidic solution. Moreover, the anti-corrosion coating was maintained in a long-term study without significant changes in the inhibition efficiency. The analysis of Arrhenius plots for uncoated and coated mild steel showed that the corrosion process is strongly endothermic when the coating is applied, resulting in high stability. Overall, the high anti-corrosion efficiency can be attributed to (i) the excellent adhesion with outstanding mechanical strength of the epoxy resin, (ii) the adhesive behavior due to the presence of electron-rich functional groups and the good mechanical properties of PDG, and (iii) the redox reversibility of PANI.

## Figures and Tables

**Table 1:** FTIR peak position and its assignment of PDG, PANI, PDG-g-PANI, and PDG-g-PANI/Al_2_O_3_.

PDG	Peak Assignment	PANI	Peak Assignment	PDG-g-PANI	Peak Assignment	PDG-g-PANI/Al_2_O_3_	Peak Assignment
3291	–OH	3241	υ NH_2_	/		/	
2915 /	υ aliphatic C–H	2930 2851	υ C-H of aniline ring	2921 2851	υ C-H of aniline ring	2917 2847	υ C–H of aniline ring
2363		2369		2370		/	
1791	υ C=O of –COOH	/		1716	υ C = O of –COOH	1716	υ C = O of –COOH
1605	υ conjugated C=C	/		/		/	
1509		1583	υ C–N of Quinoid ring	1593	υ Quinoid ring C–N	1490	υ Quinoid ring C–N
1413 / /	υ C–O	1489 1303 1170	Benzenoid ring C–N б C–H	1498 1314 1158	Benzenoid ring υ C–N б C–H	1451 1372	Benzenoid ring υ C–N & б C–H
1023	υ C–O bond	1008	–SO_3_H of DBSA	1000	υ C–O bond	1003 689 580	–SO_3_H of DBSA Al_2_O_3_ Al_2_O_3_
/		573	SO_3_^−1^ of DBSA	573	SO_3_^−1^ of DBSA	/	

**Table 2:** EDX data showing the percentage composition of PDG-g-PANI/Al_2_O_3_.

Element	Weight (%)	Atomic (%)
C	81.99	88.07
O	11.19	9.03
S	2.16	1.03
Al	4.65	1.87
Total	100	100

**Table 3:** Corrosion parameters of blank, PDG-g-PANI/Al_2_O_3_, Epoxy, and Epoxy/PDG-g-PANI/Al_2_O_3_ coated MS in 3.5% NaCl calculated from the polarization curves by Tafel extrapolation.

Coating	β Anode (V/dec)	β Cath. (V/dec)	I_corr_ (μA)	E_corr_ (mV)	Corrosion Rate (m/Year)	Inhibition Efficiency (%)
Blank MS	0.140	0.207	20.2	−777	9.224	/
Epoxy	0.300	0.099	2.16	−768	0.988	89.3
PDG-g-PANI/Al_2_O_3_	0.142	0.539	1.56	−420	0.711	92.3
Epoxy/composite	0.328	0.225	0.014	−447	0.00068	99.9

**Table 4:** Corrosion parameters of uncoated, PDG-g-PANI/Al_2_O_3_, Epoxy, and Epoxy/PDG-g-PANI/Al_2_O_3_ coated SS in 3.5% NaCl calculated from polarization curves by Tafel extrapolation.

Coating	β Anode (V/dec)	β Cath. (V/dec)	I_corr_ (μA)	E_corr_ (mV)	Corrosion Rate (m/Year)	Inhibition Efficiency (%)
Blank SS	0.444	0.163	11.90	−960	5.421	/
Epoxy	0.351	0.206	0.055	−471	0.022	99.53
Composite coated	0.535	0.463	5.72	−563	2.616	51.93
Epoxy/composite coated	0.362	0.237	0.011	−584	0.0051	99.90

**Table 5:** Corrosion parameters of uncoated, PDG-g-PANI/Al_2_O_3_, Epoxy, and Epoxy/PDG-g-PANI/Al_2_O_3_ coated SS in 1 M H_2_SO_4_ calculated from polarization curves by Tafel extrapolation.

Coating	β Anode (V/dec)	β Cath. (V/dec)	I_corr_ (μA)	E_corr_ (mV)	Corrosion Rate (m/Year)	Inhibition Efficiency (%)
Blank MS	0.159	0.209	1480	−508	675.3	/
Epoxy	0.079	0.128	2.05	−490	0.939	99.86
PDG-g-PANI/Al_2_O_3_	0.079	0.175	11.8	−475	5.370	99.2
Epoxy/Composite	0.275	0.715	0.47	−556	0.216	99.96

**Table 6:** Corrosion parameters of uncoated, PDG-g-PANI/Al_2_O_3_, Epoxy, and Epoxy/PDG-g-PANI/Al_2_O_3_ coated SS in 1 M H_2_SO_4_ calculated from polarization curves by Tafel extrapolation.

Coating	β Anode (V/dec)	β Cath. (V/dec)	I_corr_ (μA)	E_corr_ (mV)	Corrosion Rate (m/Year)	Inhibition Efficiency (%)
Blank SS	0.138	0.257	4720	−491	2157	/
Epoxy	0.131	0.193	6.65	−470	3.03	99.85
PDG-g-PANI/Al_2_O_3_	0.073	0.152	96.8	−458	44.2	97.9
Epoxy/Composite	0.211	0.239	0.0318	−490	0.014	99.99

**Table 7:** The corrosion kinetic parameters of PDG-g-PANI/Al_2_O_3_ coated MS over 41 days.

Coating Duration	β Anode (V/dec)	β Cath. (V/dec)	I_corr_ (μA)	E_corr_ (mV)	Corrosion Rate (m/Year)
Blank MS	0.140	0.207	20.2	−777	9.224
1st day	0.721	0.703	20.2 × $10^{-6}$	−407	0.00015
5th day	0.559	0.496	609 × $10^{-6}$	−384	0.00028
8th day	0.150	0.307	16.2 × $10^{-3}$	−594	0.0074
11th day	0.147	0.318	13.4 × $10^{-3}$	−522	0.0061
18th day	0.218	0.152	35 × $10^{-3}$	−681	0.0159
35th day	0.237	0.172	52 × $10^{-3}$	−679	0.0239
41st day	0.230	0.148	137 × $10^{-3}$	−713	0.0626

**Table 8:** Weight loss of uncoated and coated MS before and after immersion with the corresponding corrosion rate and inhibition efficiency.

Sample	Weight before Immersion (g)	Weight after Immersion (g)	Weight Loss (g)	Corrosion Rate (m/Year)	Inhibition Efficiency (%)
Blank MS	62.926	62.797	0.129	2.41	/
PDG-g-PANI	59.763	59.753	0.005	0.093	96.1

**Table 9:** Calculated thermodynamic parameters including apparent activation energy, entropy change, and enthalpy change in 3.5% NaCl for uncoated and Epoxy/PDG-g-PANI/Al_2_O_3_ MS.

Sample	Activation Energy (E_a_/kJ)	Enthalpy (∆H/kJ mol^−1^)	Entropy (∆S/kJ K^−1^)
Blank Mild Steel	20.5	18.3	−164.1
Epoxy/PDG-g-PANI/Al_2_O_3_ coated MS	72.7	70.3	−22.3

## Data Availability

The data presented in this study are available on request from the corresponding author.
